# Hypoxic peripheral chemoreflex stimulation‐dependent cardiorespiratory coupling is decreased in swimmer athletes

**DOI:** 10.14814/phy2.15890

**Published:** 2024-01-09

**Authors:** David C. Andrade, Alexis Arce‐Álvarez, Camila Salazar‐Ardiles, Camilo Toledo, Juan Guerrero‐Henriquez, Cristian Alvarez, Manuel Vasquez‐Muñoz, Mikel Izquierdo, Gregoire P. Millet

**Affiliations:** ^1^ Exercise Applied Physiology Laboratory, Centro de Investigación en Fisiología y Medicina de Altura (FIMEDALT), Departamento Biomedico, Facultad de Ciencias de la Salud Universidad de Antofagasta Antofagasta Chile; ^2^ Escuela de Kinesiología, Facultad de Odontología y Ciencias de la Rehabilitación Universidad San Sebastián Santiago Chile; ^3^ Navarrabiomed Hospital Universitario de Navarra (UHN), Universidad Pública de Navarra (UPNA), IdiSNA Pamplona Navarra Spain; ^4^ Laboratory of Cardiorespiratory and Sleep Physiology. Institute of Physiology. Faculty of Medicine Universidad Austral de Chile Valdivia Chile; ^5^ Centro de Investigación en Fisiología y Medicina de Altura (FIMEDALT), Departamento de Ciencias de la Rehabilitación y el Movimiento Humano, Facultad de Ciencias de la Salud Universidad de Antofagasta Antofagasta Chile; ^6^ Exercise and Rehabilitation Sciences Institute, School of Physical Therapy, Faculty of Rehabilitation Sciences Universidad Andres Bello Santiago Chile; ^7^ Dirección de Docencia de Especialidades Médicas, Dirección de Postgrado, Facultad de Medicina y Ciencias de la Salud Universidad Mayor Santiago Chile; ^8^ Institute of Sport Sciences University of Lausanne Lausanne Switzerland

**Keywords:** chemoreflex, coherence, hypoxia, swimmers

## Abstract

Swimmer athletes showed a decreased ventilatory response and reduced sympathetic activation during peripheral hypoxic chemoreflex stimulation. Based on these observations, we hypothesized that swimmers develop a diminished cardiorespiratory coupling due to their decreased hypoxic peripheral response. To resolve this hypothesis, we conducted a study using coherence time‐varying analysis to assess the cardiorespiratory coupling in swimmer athletes. We recruited 12 trained swimmers and 12 control subjects for our research. We employed wavelet time‐varying spectral coherence analysis to examine the relationship between the respiratory frequency (*R*_f_) and the heart rate (HR) time series during normoxia and acute chemoreflex activation induced by five consecutive inhalations of 100% N_2_. Comparing swimmers to control subjects, we observed a significant reduction in the hypoxic ventilatory responses to N_2_ in swimmers (0.012 ± 0.001 vs. 0.015 ± 0.001 ΔV_E_/ΔVO_2_, and 0.365 ± 0.266 vs. 1.430 ± 0.961 ΔV_E_/ΔVCO_2_/ΔSpO_2_, both *p* < 0.001, swimmers vs. control, respectively). Furthermore, the coherence at the LF cutoff during hypoxia was significantly lower in swimmers compared to control subjects (20.118 ± 3.502 vs. 24.935 ± 3.832 area under curve [AUC], *p* < 0.012, respectively). Our findings strongly indicate that due to their diminished chemoreflex control, swimmers exhibited a substantial decrease in cardiorespiratory coupling during hypoxic stimulation.

## Graphical abstract

Swimmers present a decreased ventilatory response and reduced sympathetic activation during peripheral hypoxic chemoreflex stimulation. To determine if they also present decreased cardiorespiratory coupling, we used coherence time‐varying analysis to assess the relationship between the respiratory frequency and the heart rate time series during normoxia and hypoxia. Swimmers enhibited a lower level of cardiac sympathetic respiratory coupling during hypoxic stimulation.

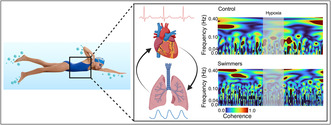

## 1 INTRODUCTION

Mainly swimming requires athletes to modulate their breathing patterns to the extent that ventilation can be interrupted for a period ranging from 1.5 to approximately 4 min (Arce‐Álvarez et al., 2021; Heusser et al., 2009). This interruption in ventilation leads to a decrease in arterial oxygen tension (PaO_2_) and an increase in arterial carbon dioxide tension (PaCO_2_) (Muth et al., 2003), which is sensed by chemoreceptors cells (Arce‐Álvarez et al., 2022; Díaz et al., 2020; Guyenet & Bayliss, 2015; Iturriaga et al., 2021). Our previous research demonstrated that prolonged breath‐holding in swimmers is associated with a notable decrease in the hypoxic ventilatory chemoreflex response compared to a control condition (Arce‐Álvarez et al., 2021). Over time, these repeated exposures to hypoxia can lead to physiological adaptations, including a blunted response of the sympathetic nervous system to hypoxia.

The carotid body (CB) houses the main peripheral chemoreceptors. This chemoreceptor organ is responsible for monitoring and detecting changes in blood chemistry, specifically the levels of oxygen (PaO_2_), carbon dioxide (PaCO_2_), pH, as well as factors such as blood flow and glucose (Arce‐Álvarez et al., 2022; Iturriaga et al., 2021; Iturriaga & Alcayaga, 2004; Kumar & Prabhakar, 2012). Activation of the chemoreceptors in the CB leads to an increase in minute ventilation (*V*_E_) and sympathetic drive to meet metabolic demands (Iturriaga et al., 2016). In normal physiological functioning, respiratory activity and sympathetic nervous system activity are closely linked (Moraes et al., 2012). Changes in respiratory patterns, such as breathing rate and depth, can influence sympathetic nervous system activity and vice versa. This coupling is crucial for maintaining respiratory control and cardiovascular function.

Consequently, as swimmers exhibit decreased ventilatory chemoreflex responses and, therefore, sympathetic drive (as indicated by an increase in the low‐frequency component of heart rate variability) (Arce‐Álvarez et al., 2021), there may be a coupling between cardiac‐mediated sympathetic control and ventilation in these athletes. However, this relationship has yet to be described in swimmers. Accordingly, it is possible to hypothesize that swimmers showed a decreased hypoxic peripheral chemoreflex stimulation‐dependent cardiorespiratory coupling. Then, we sought to determine the cardiorespiratory coupling in swimmer athletes using wavelet coherence time‐varying analysis.

## 2 MATERIALS AND METHODS

### 2.1 Ethical statements

Protocols were approved by the Ethical Committee of the Universidad Mayor (Approval number #169_2019) and were performed according to the Declaration of Helsinki. Participants were carefully informed about the experimental procedures and the possible risks and benefits associated with their participation in the study. Written informed consent was obtained from participants.

### 2.2 Subjects

Twelve young trained regional‐ to national‐level competitive swimmers (7 males and 5 females; age, 19.7 ± 2.2 years; height, 171.6 ± 5.3 cm; body mass, 68.3 ± 7.9 kg; body mass index [BMI], 23.4 ± 2.8 kg/m^2^), with 5–12 years of swimming training, and a mean weekly training volume of ~4 h per day, five times per week, participated in this study. Twelve controls (5 males and 7 females; age, 21.3 ± 3.2 years; height, 168.9 ± 4.2 cm; body mass, 65.2 ± 3.6 kg; BMI, 23.1 ± 2.0 kg/m^2^) also volunteered to participate in this study. All females were assessed (by a female technician) during the early follicular phase of their menstrual cycle. Experiments were conducted between 08:00 and 17:00 h. Forty‐eight hours before experiments, participants were asked to avoid consuming alcohol, cigarettes, caffeine, or drugs that may alter autonomic control. None of the participants were taking any medication or had a personal or family history of any cardiac, ventilatory, or endocrine disorder.

### 2.3 Experimental design

A descriptive cross‐sectional study was performed to determine time‐varying coherence and ventilatory response to hypoxia in young, highly trained swimmers compared to controls. The inclusion criteria were (i) high‐performance swimmers with <12 years of training; (ii) from national or university teams, active participants in national or international competitions; and a minimum of 20 h of training per week. Exclusion criteria were (i) potential medical problems or history of cardiorespiratory diseases; (ii) any cardiovascular or respiratory surgery in the past 2 years; (iv) being in the course of an acute illness or consumption of any drug or pharmacological ergogenic aid; and (v) history of chronic obstructive or restrictive pulmonary diseases.

On the first day, body mass, height, and resting metabolic rate (RMR) were assessed. Body mass was estimated to the nearest 0.1 kg using a digital scale (BF‐350, Tanita, IL, USA). Height was measured using a wall‐mounted stadiometer (HR‐200, Tanita, Japan) and recorded to the nearest 0.1 cm. The BMI was calculated as kg/m^2^. The RMR was determined through indirect calorimetry (Quark CPET metabolic cart; COSMED) (see section 2.7).

On the second day, the participants were instrumented and positioned supine at an ambient temperature of ~22°C. Instrumentation includes: a 2‐lead electrocardiogram (ECG), core temperature regulator, and an orofacial mask (Hans Rudolph3, 3700A) connected to a gas mixing chamber to measure the airflow and expired gases. From respiratory flow, tidal volume (*V*_T_) was calculated (FE141, ADInstruments Inc). From *V*_T_ and respiratory frequency (*R*_f_), minute ventilation was calculated (*V*_E_ = *V*_T_*R_f_). The expired fraction of end‐tidal carbon dioxide (F_E_CO_2_), end‐tidal oxygen (F_E_O_2_) levels, and fraction inspired of O_2_ (F_I_O_2_) were calculated from CO_2_ and O_2_ gas analyzers (ML206, ADInstruments Inc). After instrumenting the subjects, they were given a 15‐min rest period in a supine position before the recording started. After this period, baseline parameters were recorded for 20 min. In addition, they were instrumented with eye masks and headphones to reduce external noise (MPA 101, Masprot, Chile) to blunt the effect of the manipulation of gases on participants' arousal. Ventilatory data and gas exchanges were digitized using PowerLab Data Acquisition System (PowerLab, 16SP, ADInstruments Inc) and analyzed with LabChart 8.0 (ADInstruments Inc).

### 2.4 Hypoxic ventilatory response (HVR)

HVR was evaluated by a poikilocapnic transient hypoxic challenge as previously described (Pfoh et al., 2016). Participants underwent three consecutive trials (each trial was separated by 5 min) with five breaths of 100% N_2_. N_2_ was blended into a port on the mask through N_2_ tubing. After applying N_2_, 15 min elapsed until ventilatory parameters returned to baseline levels. This experiment was performed with a PowerLab Data Acquisition System (PowerLab, 16SP, ADInstruments Inc).

### 2.5 Electrocardiogram (ECG)

The 2‐lead ECG was recorded using a bio‐amplifier connected to a digital recording system (PowerLab, 16SP, ADInstruments Inc). The electrodes (3M, St. Paul, MN) were placed in the second derivative (DII) from the Einthoven triangle with participants in a supine position. The ECG and breathing gases, and ventilation were recorded continuously in all experiments considering the peripheral chemoreflex test. The sampling frequency was set at 4 kHz and was amplified x100. The ECG analysis was performed with L LabChart 8.0 (ADInstruments Inc).

### 2.6 Wavelet time‐varying coherence analysis

To determine the coherence between signals, the squared coherence magnitude (De Boer & Karemaker, 2019; Keissar et al., 2009), which corresponds to a measure of the correlation between pairs of X‐Y signals (i.e., cardiac and respiratory time series) in the time‐frequency domain was used, following previous recommendations (Xu et al., 2020). Using a Morlet wavelet, $\Psi \left(t\right)$, defined as:

$$\Psi \left(\frac{t}{s}\right)=\frac{1}{\pi^{4}}e^{-\frac{1}{2}{\left(\frac{t}{s}\right)}^{2}}e^{j2\pi f_{0}t}$$

where $j=\sqrt{-1}$, $f_{0}$ corresponds to the center frequency of $\Psi \left(t\right)$, and s as its scale. Subsequently, the continuous wavelet transforms function of a discrete signal of N‐samples with $x_{n}$ sequences, with a ∆t period, is denoted by

WnS=∑i=0N−1xiΨ*i−n∆ts=∑i=0N−1Xi∆tsΨ0*i−n∆ts

where * denotes the complex conjugate. Then, the wavelet power density estimator is defined as

$$W_{n}^{\mathrm{xx}}=W_{n}^{x}\left(s\right)W_{n}^{x}*\left(s\right)$$

From the wavelet transform of two sequence signals $x_{n}$ and $y_{n}$, the cross‐wavelet transform, which identifies areas of high‐power magnitude in common, is defined by

$$W_{n}^{\mathrm{xy}}=W_{n}^{x}\left(s\right)W_{n}^{y}*\left(s\right)$$

Finally, the squared coherence estimator of the wavelet transform ($R^{2}$) of two signals is the coherence of the two signals in the time‐frequency domain. $R^{2}$ is interpreted as a localized correlation coefficient in the time‐frequency domain, which ranges from 0 to 1, and is computed as the smoothed squared magnitude of the cross‐wavelet transform normalized by the power spectral wavelet of X and Y:

$$R^{2}\left(s\right)=\frac{{\left|S(W_{n}^{\mathrm{xy}}\left(s\right)s^{-1}\right|}^{2}}{S\left(\left|W_{n}^{\mathrm{xx}}\left(s\right)\right|s^{-1}\right)S\left(\left|W_{n}^{\mathrm{yy}}\left(s\right)\right|s^{-1}\right)}$$

To determine a threshold of significant coherence between the signals, 500 pairs of surrogate data as the 90th percentile of the coherence sampling distribution at each scale/frequency through the Monte Carlo method was obtained from the wavelet transform coherence (Grinsted et al., 2004). Three frequency bands were analyzed: very low frequency (VLF, 0.0–0.04 Hz), low frequency (LF, 0.04–0.1 Hz), and high frequency (HF, 0.1–0.4 Hz). All analyses were performed with Matlab Software (v. R2021b, The MathWorks, Inc).

For validation proposes, a previously published wavelet coherence analysis Matlab routine was implemented (Grinsted et al., 2004). This algorithm produces a distribution of coherence values under the null hypothesis that no association exists between the series. Furthermore, it performs a significance test through a Monte Carlo simulation, giving statistical significance to the coherence values. Moreover, following the recommendations of Keissar et al. (2009) and Garg et al. (2013), the standard deviation was estimated to establish the statistical validity of the coherence estimation between signals per band. Average standard deviation values equal to or less than 0.2 were obtained, which agrees with previous evidence (Garg et al., 2013; Keissar et al., 2009).

### 2.7 Resting metabolic rate

Resting metabolic rate (RMR) was determined by indirect calorimetry, as previously described (Speakman & Selman, 2003). All participants were instructed to come to the indirect calorimetry lab between 8 and 10 am after fasting for at least 12 h and abstaining from strenuous physical activity or consuming alcohol, caffeine, and stimulant substances during the 24 h prior to the measurement (Abulmeaty et al., 2020; Compher et al., 2006; Nieman et al., 2013; Robles‐González et al., 2021; Speakman & Selman, 2003). During RMR measurement, participants breathed through an oronasal mask (7450 Series Silicone V2, Hans Rudolph) for expired gas collection and analysis (Quark CPET metabolic cart; COSMED). Before experiments, the device was warmed up and calibrated before its first use and recalibrated every three measurements with a known calibration gas (O_2_ 15%, CO_2_ 5%, N_2_ balanced) (Abulmeaty et al., 2020; Nieman et al., 2013). The RMR measurement was performed in a specially conditioned room isolated from noise at 23°C and 50% humidity. Before the measurement, the participant took a rest of 30 min. The subjects were instrumented and placed supine during 40 min of measurement. From the total recording, the first 5 min were discarded as part of the acclimatization period, and the respiratory quotient (RQ), protein oxidation, carbohydrates, and lipids were calculated from the remaining 35 min. Protein oxidation, carbohydrates, and lipids were expressed as % of total RMR. The recording and analysis were performed with OMNIA, Cardiopulmonary Diagnostic Suite v 1.4 (Quark CPET metabolic cart; COSMED).

### 2.8 Statistical analysis

Data were expressed as mean ± standard deviation (SD). The normality of the data was assessed using the Shapiro–Wilk test, and Levene's test determined the homoscedasticity of the variance. Differences between groups were evaluated using unpaired *t*‐tests for variables with normal distribution and Mann–Whitney test for variables with non‐normal distribution. For the analysis between normoxia and hypoxia, the data between both groups were analyzed using a two‐way ANOVA followed by the Holm–Sidak post hoc test. (GraphPad Prism software Inc., version 8.0). Significant differences were set at *p* < 0.05.

## 3 RESULTS

### 3.1 Baseline parameters

Baseline parameters are shown in Table 1. No significant differences were observed between the swimmers and control groups in respiratory, cardiovascular, and autonomic control at baseline, except for F_i_CO_2_ and *T*_E_ (*p* < 0.05, Table 1), which showed significant differences (*p* < 0.05, Table 1). Swimmers exhibited a significant increase in FiCO_2_ and *T*_E_ compared to the control group (Table 1). The baseline metabolic evidenced a significant difference in RMR and relative CHO oxidation in swimmers compared to the control condition (*p* < 0.05, Table 1). The relative FAT oxidation was slightly increased in swimmers relative to control but was not significantly different (Table 1).

**TABLE 1:** Basal anthropometric, respiratory, cardiovascular, autonomic, and metabolic characteristics of swimmers compared to control participants at rest condition.

	Control (*n* = 12)	Swimmers (*n* = 12)
Respiratory		
*R*_f_ (breaths/min)	14.66 ± 2.92	15.75 ± 3.64
*V*_T_ (mL)	638 ± 195	624 ± 126
*V*_E_ (L/min)	8.84 ± 1.97	9.15 ± 2.40
VO_2_ (mL/min)	312.00 ± 56.63	313.65 ± 81.63
VCO_2_ (mL/min)	263.00 ± 52.30	255.52 ± 57.00
RQ	0.85 ± 0.08	0.83 ± 0.10
O_2_exp (mL)	102.79 ± 32.81	102.61 ± 23.23
CO_2_exp (mL)	27.36 ± 8.51	24.67 ± 5.19
PetO_2_ (mmHg)	97.98 ± 4.89	94.44 ± 19.02
PetCO_2_ (mmHg)	36.72 ± 2.77	34.66 ± 2.99
F_i_O_2_ (%)	20.46 ± 0.19	20.60 ± 0.22
F_i_CO_2_ (%)	0.37 ± 0.11	0.19 ± 0.07*
SpO_2_ (%)	97.54 ± 1.27	97.56 ± 0.81
*T*_I_ (s)	3.20 ± 5.08	1.80 ± 0.46
*T*_E_ (s)	2.51 ± 0.59	2.30 ± 0.66*
*T*_TOT_ (s)	5.71 ± 4.99	4.10 ± 1.11
Cardiovascular		
HR (beats/min)	64.69 ± 10.09	70.69 ± 9.93
R‐R (s)	956.54 ± 152.18	870.88 ± 117.18
Heart rate variability (HRV)		
LF (n.u.)	25.58 ± 16.98	24.67 ± 9.39
HF (n.u.)	72.10 ± 15.63	74.14 ± 13.10
LF/HF	0.43 ± 0.40	0.40 ± 0.40
Metabolic		
RMR (kcal/day)	1986.62 ± 332.06	2277.00 ± 339.65*
FAT (%)	51.94 ± 24.09	68.53 ± 16.88
CHO (%)	48.06 ± 24.09	28.98 ± 10.94*

*Note*: Values are means ± SD. Data with non‐normal distribution were analyzed using nonparametric Mann–Whitney test. Data with normal distribution were analyzed using a parametric unpaired *t*‐test.

Abbreviations: CHO, relative to RMR carbohydrates oxidation; FAT, relative to RMR fat oxidation; F_i_CO_2_, concentration of inspired carbon dioxide; F_i_O_2_, concentration of inspired oxygen; HF, high‐frequency component of HRV; HR, heart rate; LF, low‐frequency component of HRV; PetCO_2_, end‐tidal CO_2_ pressure; PetO_2_, end‐tidal oxygen pressure; *R*_f_, respiratory frequency; RMR, resting metabolic rate; RQ, respiratory quotient; R‐R, time between ECG peak R; SpO_2_, oxygen saturation; *T*_E_, expiratory time; *T*_I_, inspiratory time; *T*_TOT_, total time of one breath; VCO_2_, carbon dioxide production; *V*_E_, ventilation; VO_2_, oxygen uptake; *V*_T_, tidal volume.

* *p* < 0.05 versus control group.

### 3.2 Hypoxic chemoreflex function

The breathing and metabolic responses to hypoxia are shown in Table 2. In normoxia, there were no significant differences between swimmers and controls in R_F_ (*p* > 0.05) (Table 2). However, during hypoxia, both groups showed significant *V*_T_, VO_2_, and VCO_2_ increases and decrement SpO_2_ (*p* < 0.05, Table 2). Furthermore, the data did not indicate significant between‐group effects (**∆**normoxia‐hypoxia) in **∆**R_f_, **∆**V_T,_ **∆**VO_2_, **∆**VCO_2_, and SpO_2_ (Table 2).

**TABLE 2:** Cardiovascular and respiratory responses to severe hypoxic challenge in swimmers and control participants.

	Controls (*n = 12*)		Swimmers (*n = 12*)		Controls	Swimmers
	Normoxia	Hypoxia	Normoxia	Hypoxia	∆ Normoxia ‐ hypoxia	∆ Normoxia ‐ hypoxia
*R*_f_ (breaths/min)	14.34 ± 2.87	14.58 ± 3.20	11.83 ± 3.63	11.75 ± 4.37	0.24 ± 1.51	−0.08 ± 2.14
*V*_T_ (mL)	707.42 ± 237.29	1129.58 ± 415.20*	810.58 ± 398.13	1240.92 ± 507.20*	422.17 ± 350.45	430.33 ± 386.60
*V*_T_ (mL/kg)	11.42 ± 3.50	18.57 ± 7.02*	11.01 ± 4.64	11.29 ± 7.90*	7.15 ± 5.86	6.64 ± 5.13
VO_2_ (mL/kg/min)	4.94 ± 2.55	18.05 ± 9.27*	4.52 ± 1.97	16.51 ± 7.43*	13.12 ± 7.30	11.99 ± 5.79
VCO_2_ (mL/kg/min)	4.25 ± 2.04	6.95 ± 3.16*	3.91 ± 1.68	5.58 ± 1.84*	2.03 ± 1.29	1.77 ± 1.60
SpO_2_ (%)	98.47 ± 0.52	88.93 ± 4.45*	98 ± 0.00	86.51 ± 2.55*	−9.08 ± 5.62	−11.20 ± 5.29

*Note*: Values are means ± SD. Unpaired *t*‐test and Mann–Whitney *U*‐test were used to parametric and nonparametric variables, respectively. Two‐way ANOVA with repeated measures followed by Holm–Sidak post hoc test was performed.

Abbreviations: *R*_f_, respiratory frequency; SpO_2_, Oxygen saturation; VO_2_, oxygen consumption; *V*_T_, tidal volume; VCO_2_, carbon dioxide production.

* *p* < 0.05 versus normoxia.

**FIGURE 1:**
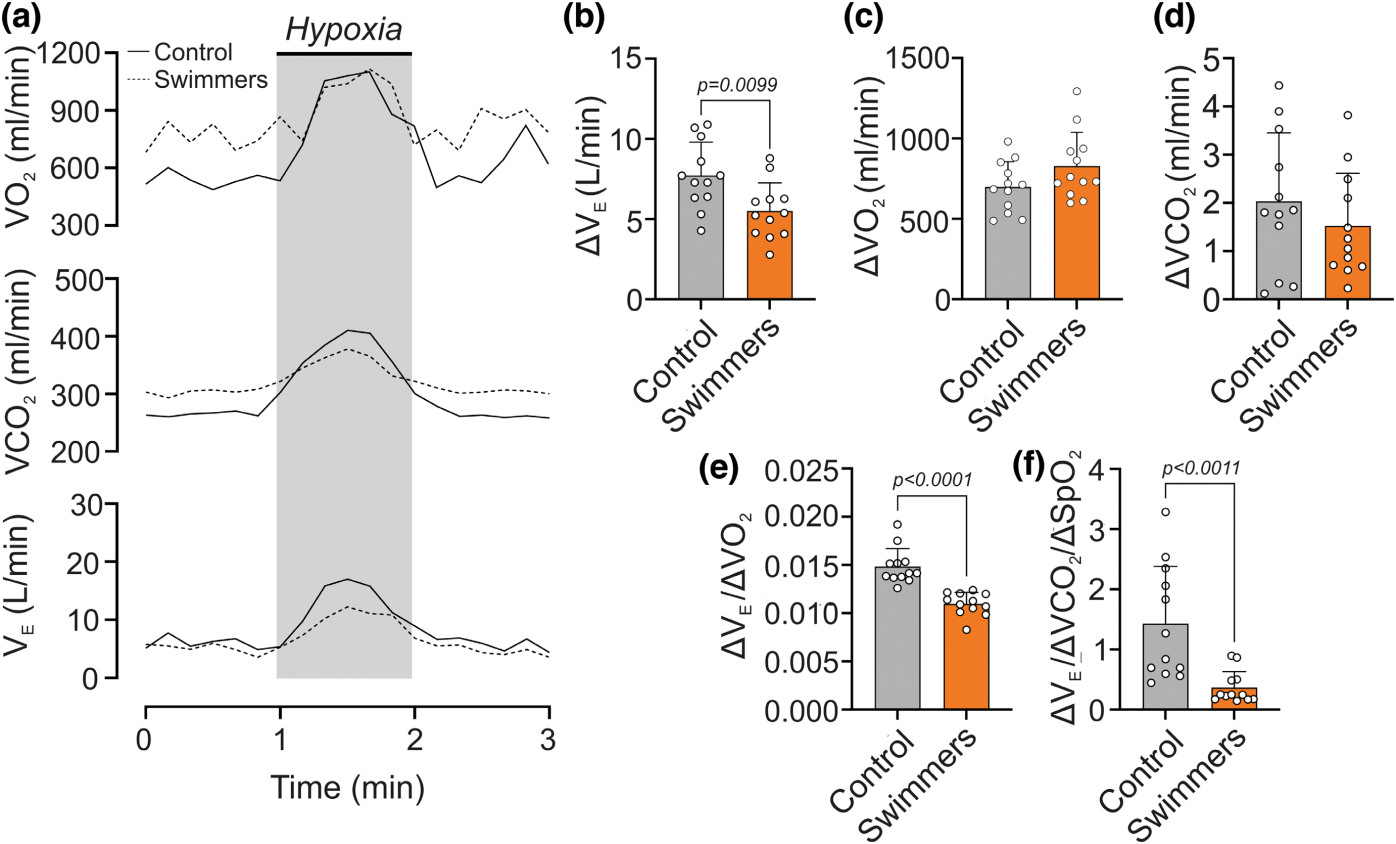
Hypoxic ventilatory responses in young trained swimmers (HVR). (a) Representative minute ventilation (*V*_E_), oxygen uptake (VO_2_), and carbon dioxide production (VCO_2_) recording of one control and swimmer participant during a hypoxic challenge (pure N_2_). Note that swimmers displayed a decreased *V*_E_ response compared to the control participants. (b) Quantification of ΔV_E_ between normoxia and hypoxia in both groups. (c) Quantification of ΔVO_2_ between normoxia and hypoxia in both groups. (d) Quantification of ΔVCO_2_ between normoxia and hypoxia in both groups. (e, f) The swimmer's group showed a significantly lower HVR through ΔV_E_/ΔVO_2_ and ΔV_E_/ΔVCO_2_/ΔSpO_2_, compared to the controls. Unpaired *t*‐test for (b, c, e, and f); and Mann–Whitney test for (d). Values are mean ± SD; control, *N* = 12, and swimmers, *n* = 12.

*Hypoxic ventilatory response*. Swimmer athletes showed a significant decrease of ΔV_E_ (*p* < 0.001) compared to the control group, without significant differences in ΔVO_2_ and ΔVCO_2_ during hypoxic stimulation (Figure 1a–d). Indeed, swimmers showed a markedly lower ΔV_E_ response than the controls (5.50 ± 1.75 vs. 7.72 ± 2.08 L/min, respectively) (Figure 1b). Consequently, the HVR expressed as ΔV_E_/ΔVO_2_ and as ΔV_E_/ΔVCO_2_/ΔSpO_2_ was significantly lower in swimmers compared to the control participants (0.012 ± 0.001 vs. 0.015 ± 0.001 ΔV_E_/ΔVO_2_, *p* < 0.001, respectively) (0.365 ± 0.266 vs. 1.430 ± 0.961 ΔV_E_/ΔVCO_2_/ΔSpO_2_, *p* < 0.001, respectively) (Figure 1e,f).

### 3.3 Wavelet time‐varying coherence

**FIGURE 2:**
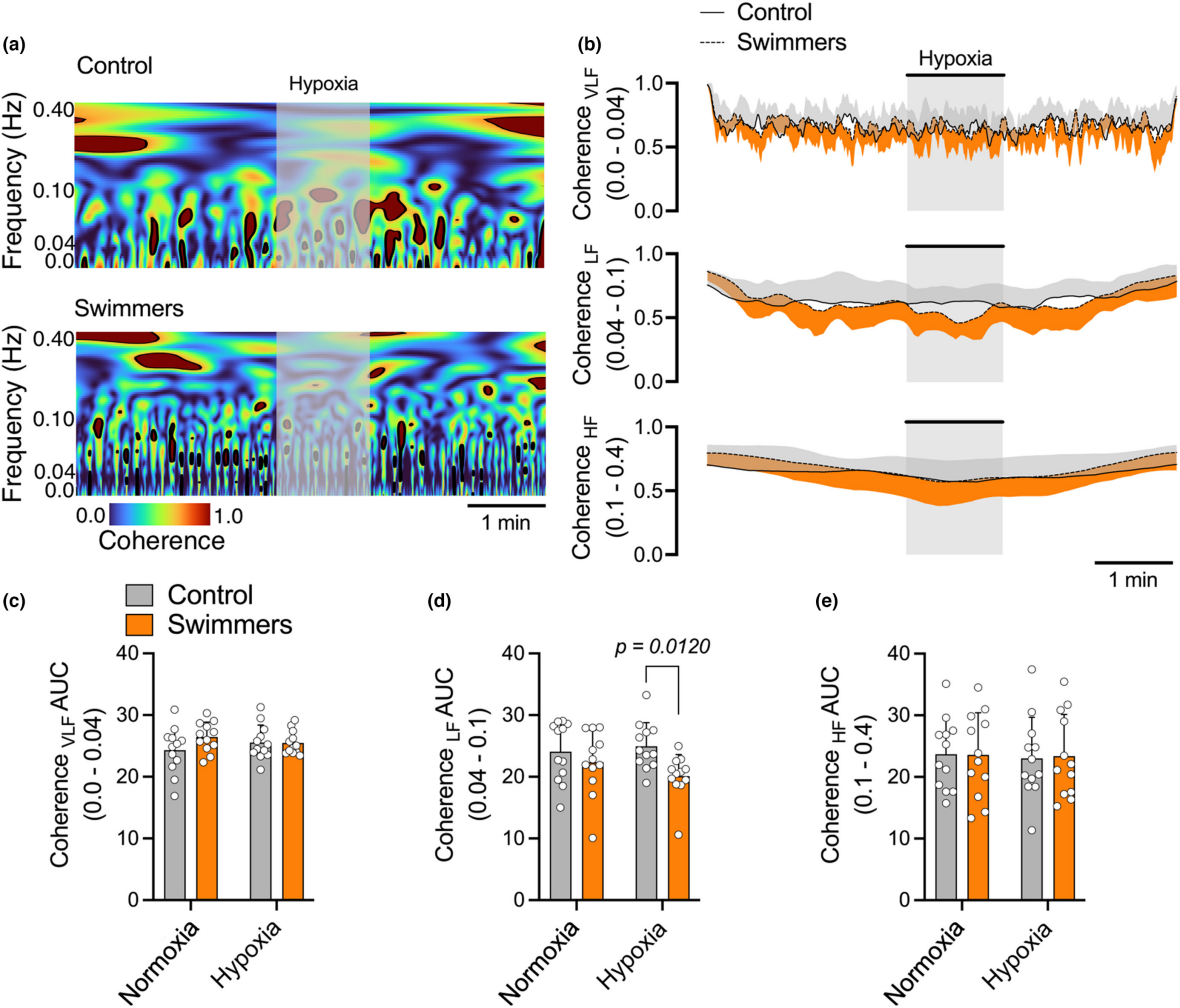
Time‐varying coherence analysis in normoxia and hypoxia in young trained swimmers. (a, b) Representative data of VLF, LF, and HF cutoff coherence analysis during normoxia and hypoxia (pure N_2_) of one control and swimmer participant. (c, d, e) Quantification of VLF, LF, and HF cutoff coherence, respectively, between normoxia and hypoxia in both groups. Note that swimmers displayed a decrease in LF cutoff in hypoxic challenge response compared to the control participants. Two‐way ANOVA with repeated measures followed by Holm–Sidak post‐hoc test. Values are mean ± SD; control *N* = 12, and swimmers, *n* = 12.

A time‐varying coherence analysis was conducted during both normoxia and hypoxic stimulation The coherence values in the VLF and HF ranges did not show significant differences between swimmers and the control group in both normoxia and hypoxic stimulation conditions (*p* > 0.05) (Figure 2a‐c,e). However, during hypoxic stimulation, a notable reduction in coherence was observed in swimmers compared to control subjects at the low frequency (LF) cutoff frequency range (0.04–0.1 Hz) (20.118 ± 3.502 vs. 24.935 ± 3.832 AUC, *p* < 0.012, respectively) (Figure 2a,b,d).

## 4 DISCUSSION

The main aim of the present study was to determine the cardiorespiratory coupling in swimmer athletes using wavelet coherence time‐varying analysis. Based on our hypothesis, we anticipated that the decreased peripheral chemoreflex drive observed in swimmers would be associated with a reduction in hypoxic‐dependent cardiac‐related sympathetic respiratory coupling. The key findings of our study were as follows. When compared to the control group, swimmers exhibited the following characteristics: (i) a significant decrease in their respiratory response to hypoxia; (ii) diminished hypoxic peripheral chemoreflex drive; and (iii) a lower level of cardiorespiratory coupling during hypoxic stimulation. These results support the notion that the reduced ventilatory response to hypoxia, which aligns with our previous findings (Arce‐Álvarez et al., 2021), may contribute to a decreased cardiorespiratory coupling under hypoxic conditions.

### 4.1 Hypoxic ventilatory response in highly trained swimmers

The ventilatory response to hypoxia mainly depends on the CB glomus cells, which respond to decreased O_2_ arterial tension. Although it is well known that the CB is the primary sensor of arterial oxygen, the mechanism by which CB glomus cells sense is still being studied. However, the more accepted model is that hypoxia inhibits the background K^+^‐channels, leading to the influx of Ca^2+^ into the glomus cell, causing its depolarization and release of excitatory neurotransmitters, causing the cardiorespiratory responses to hypoxia. Aquatic immersion sports are characterized by prolonged breath‐holding (Heusser et al., 2009), eliciting changes in arterial oxygen tension (PaO_2_) and arterial carbon dioxide tension (PaCO_2_) during apneas (Muth et al., 2003). These changes can stimulate chemoreceptors (Dempsey et al., 2012; Guyenet & Bayliss, 2015; Kumar & Prabhakar, 2012) and contribute to apnea termination. In line with our previous findings (Arce‐Álvarez et al., 2021, 2022), we observed a decrease in chemoreflex drive in swimmers, indicating that they adapted to their sport's respiratory challenges. Similarly, the current study demonstrates that swimmer athletes also exhibit a reduced ventilatory response to hypoxia. Specifically, swimmers displayed a decreased hypoxic ventilatory response (HVR) when stimulated by a pure nitrogen (N_2_) challenge, which confirms our earlier observations (Arce‐Álvarez et al., 2021). Thus, long‐term underwater hypoxia training could desensitize the background K^+^ channels, producing a delayed release of excitatory neurotransmitters, which allows the swimmers to maintain more extended periods of breath‐holding than a control subject.

In contrast, studies involving breath‐holding divers and control participants have reported no significant differences in hypoxic‐dependent breathing responses (Breskovic et al., 2010; Dujic et al., 2008). Similarly, Bruce et al. (2021) found that the ventilatory response to hypoxia was not associated with chemoreflex drive. However, it is essential to note that our results differ from these findings due to several factors. First, our study focused on young‐trained swimmers rather than adults, which may influence the physiological adaptations observed in response to underwater training. Additionally, the specific characteristics and demands of aquatic immersion sports may contribute to the differences in respiratory responses between swimmers and other groups. Overall, our findings highlight the unique respiratory adaptations exhibited by young‐trained swimmers and emphasize the importance of considering the specific population and training context when interpreting the results of respiratory studies in aquatic sports.

### 4.2 Cardiorespiratory coupling during hypoxia in highly trained swimmers

As previously mentioned, swimmers can withstand breath‐holding, associated with hypoxic hyporeflexia (Arce‐Álvarez et al., 2021, 2022). During breath‐holding, the autonomic response is crucial in ensuring adequate blood flow and oxygen supply to active muscles (Breskovic et al., 2010; Heusser et al., 2009). Hypoxemia during apnea can trigger a positive cardiac chronotropic response mediated by sympathoexcitation, independent of the preceding inflation reflex (Siebenmann et al., 2019). In line with our previous findings (Arce‐Álvarez et al., 2021), we observed that swimmers exhibited an increase in low‐frequency heart rate variability, primarily influenced by the sympathetic nervous system (Goldstein et al., 2011). This was accompanied by a positive chronotropic response during a pure nitrogen (N_2_) challenge.

As swimmers demonstrate a decreased chemoreflex drive and a modest heart rate response during hypoxic stimulation, we aimed to investigate the potential sympathetic‐dependent cardiorespiratory coupling during a pure N_2_ challenge. Our current results indicate that swimmers exhibited reduced cardiorespiratory coupling in response to hypoxia. This finding may be attributed to their diminished chemoreflex drive and attenuated sympathetic‐related cardiac response to hypoxia. Such characteristics may provide swimmers with greater resistance to hypoxic challenges, thereby enhancing the efficiency of their cardiorespiratory system during these challenging events.

The present study has certain limitations that should be acknowledged. First, all our experiments were conducted at rest, and we did not replicate the conditions of exercise or immersion in water, which are the natural environments for swimmers. Therefore, the findings of our study may only partially reflect the physiological responses that occur during swimming activities. Future research should investigate cardiorespiratory coupling in swimmers during exercise or in water to better understand their autonomic and respiratory responses in their specific context. Additionally, we focused solely on the peripheral chemoreflex response. We did not assess the contribution of the central chemoreceptors to the ventilatory response to CO2. We did not control end‐tidal CO_2_ pressure; hence, we used a poikilocapnic hypoxic stimulation. Thus, future studies need to investigate both peripheral and central chemoreceptor responses to hypoxic and hypercapnic stimuli and end‐tidal CO_2_ pressure to explore whether cardiorespiratory coupling is also observed during the stimulation of central chemoreceptors with controlled CO_2_. This would provide valuable insights into the integrated control mechanisms of the respiratory and autonomic systems in swimmers and further enhance our understanding of their physiological adaptations. By addressing these limitations, future investigations can provide a more comprehensive and detailed understanding of the complex interplay between chemoreflex responses, sympathetic‐mediated cardiac activity, and respiratory control in swimmers.

## 5 CONCLUSIONS

Respiratory and sympathetic nervous system activity are intricately interconnected in normal physiological conditions, whether at rest or during exercise. Any alterations in respiratory patterns can impact sympathetic‐related cardiac system activity and vice versa. This mutual relationship is crucial for maintaining optimal respiratory control and cardiovascular function. When there is an increased demand for respiratory efforts, such as during exercise or exposure to low oxygen levels (hypoxia), it is typical to observe heightened sympathetic nervous system activity to support the heightened respiratory demands. This heightened sympathetic outflow can result in changes in heart rate, blood pressure, and other cardiovascular responses. Conversely, sympathetic activation can also influence respiratory function. Our data strongly indicate that, owing to decreased chemoreflex control, swimmers significantly reduced sympathetic‐related cardiorespiratory coupling during hypoxic stimulation.

## AUTHOR CONTRIBUTIONS

David C. Andrade and Alexis Arce‐Álvarez performed data collection and analysis, performed interpretation of the data, and contributed to the preparation of the manuscript. Juan Guerrero‐Henriquez performed data analysis. Camila Salazar‐Ardiles, Camilo Toledo, Juan Guerrero‐Henriquez, Cristian Alvarez, Manuel Vasquez‐Muñoz, Mikel Izquierdo, and Gregoire P. Millet contributed to the preparation of the manuscript. David C. Andrade contributed to the concept of the project. All data analysis and interpretation were undertaken in the laboratory of David C. Andrade All authors approved the final version of the manuscript.

## CONFLICT OF INTEREST STATEMENT

None of the authors declare competing financial interests.

## Data Availability

The datasets are available from the corresponding author upon reasonable request.
